# *Aspergillus niger* as a cell factory for the production of pyomelanin, a molecule with UV-C radiation shielding activity

**DOI:** 10.3389/fmicb.2023.1233740

**Published:** 2023-07-20

**Authors:** Stella Marie Koch, Carsten Freidank-Pohl, Oliver Siontas, Marta Cortesao, Afonso Mota, Katharina Runzheimer, Sascha Jung, Katarina Rebrosova, Martin Siler, Ralf Moeller, Vera Meyer

**Affiliations:** ^1^Radiation Biology Department, Aerospace Microbiology Research Group, German Aerospace Center, Institute of Aerospace Medicine, Cologne, Germany; ^2^Applied and Molecular Microbiology, Institute of Biotechnology, Technische Universität Berlin, Berlin, Germany; ^3^Department of Microbiology, Faculty of Medicine, Masaryk University (MUNI) and St. Anne's Faculty Hospital, Brno, Czechia; ^4^Institute of Scientific Instruments of the Czech Academy of Sciences, Brno, Czechia

**Keywords:** *Aspergillus niger*, melanin, pyomelanin, radioprotection, cosmic radiation, space exploration

## Abstract

Melanins are complex pigments with various biological functions and potential applications in space exploration and biomedicine due to their radioprotective properties. Aspergillus niger, a fungus known for its high radiation resistance, is widely used in biotechnology and a candidate for melanin production. In this study, we investigated the production of fungal pyomelanin (Pyo_Fun_) in *A. niger* by inducing overproduction of the pigment using L-tyrosine in a recombinant Δ*hmgA* mutant strain (OS4.3). The Pyo_Fun_ pigment was characterized using three spectroscopic methods, and its antioxidant properties were assessed using a DPPH-assay. Additionally, we evaluated the protective effect of Pyo_Fun_ against non-ionizing radiation (monochromatic UV-C) and compared its efficacy to a synthetically produced control pyomelanin (Pyo_Syn_). The results confirmed successful production of Pyo_Fun_ in *A. niger* through inducible overproduction. Characterization using spectroscopic methods confirmed the presence of Pyo_Fun_, and the DPPH-assay demonstrated its strong antioxidant properties. Moreover, Pyo_Fun_ exhibited a highly protective effect against radiation-induced stress, surpassing the protection provided by Pyo_Syn_. The findings of this study suggest that Pyo_Fun_ has significant potential as a biological shield against harmful radiation. Notably, Pyo_Fun_ is synthesized extracellularly, differing it from other fungal melanins (such as L-DOPA- or DHN-melanin) that require cell lysis for pigment purification. This characteristic makes Pyo_Fun_ a valuable resource for biotechnology, biomedicine, and the space industry. However, further research is needed to evaluate its protective effect in a dried form and against ionizing radiation.

## 1. Introduction

Crewed space missions to the Moon and Mars are the main goal of various space-faring nations. However, sending humans farther into space requires that they not only have sufficient oxygen, food, medicine, clean water, and circular waste disposal systems available during their missions but also that they are adequately protected from cosmic and solar radiation. While the Earth's magnetic field provides this protection for us humans on Earth, space travelers must be shielded from radiation by the outer casing of spacecraft or spacesuits (Norbury et al., 2019). Fungal biotechnology holds great potential for significantly contributing to the manifold efforts required to enable long-distance space travel, lunar or Martian habitation, and ultimately, Earth-independent space missions (Cortesão et al., 2020b). The filamentous fungal cell factory *Aspergillus niger* is of special interest in this endeavor due to several factors: (i) It is a companion of healthy humans, belonging to their mycobiota (Peters et al., 2017), which is probably one of the reasons why *A. niger* is prevalent in the indoor environment of the international space station ISS (Romsdahl et al., 2018). (ii) It is one of the main microbial cell factories applied by Earth's biotechnology due to *A. niger*'s ability to efficiently transform renewable plant biomass into a wide product spectrum (e.g., organic acids, proteins, enzymes, and secondary metabolites), which, in turn, lead to the production of food, pharmaceuticals, textiles, and biofuels (Cairns et al., 2018). (iii) It grows well under conditions simulating microgravity (Cortesão et al., 2022). (iv) Its spores are extremely resistant to space radiation including UV-C (254 nm), cosmic radiation (helium and iron ions), and X-ray radiation (Cortesão et al., 2020b).

Many pigments, particularly melanins, are known to shield fungi, including *A. niger*, against radiation-induced stress and reactive oxygen species via absorption, scattering, free radical scavenging, and their key role in intracellular DNA protection (Cairns et al., 2018; Cortesão et al., 2020b; Vasileiou and Summerer, 2020). Melanins are complex polymers with great multifunctionality, as they fulfill many essential intracellular functions in distinct species of all biological niches, such as UV light and oxidative stress protection, energy transduction, chelation of heavy metals, and more. Notably, several diverse kinds of melanins (e.g., allomelanin, eumelanin, neuromelanin, pheomelanin, and pyomelanin) which have been classified based on the precursors used for their biosynthesis are found in bacteria, fungi, plants, and animals which also indicates their significant evolutionary role and have been classified based on the precursors used for their biosynthesis (Cao et al., 2021). We are, however, only beginning to understand exactly how melanin-driven cellular protection works and which kind of melanins are being produced in which fungus (Singh et al., 2021; Gao et al., 2022). Pyomelanin is of exceedingly high interest for future applications because of its superior antioxidative and radical scavenging properties, its non-toxicity, and hyperthermostability (Lorquin et al., 2021).

It is widely accepted that fungi synthesize melanin either by a polyketide-based biosynthetic route that starts off with acetyl-CoA and malonyl-CoA and eventually polymerizes 1,8-dihydroxynaphthalene to DHN-melanin (= allomelanin), or through L-tyrosine and eventual polymerization of L-3,4-dihydroxyphenylalanine toward L-DOPA-melanin (= eumelanin). However, as described earlier for *A. fumigatus* (Schmaler-Ripcke et al., 2009), pyomelanin can also be formed as a product of the L-tyrosine/L-phenylalanine degradation pathway, whereby homogentisic acid (HGA) is formed which auto-oxidizes to the brownish (Tokuhara et al., 2018) pyomelanin precursor 1,4-benzoquinone acetic acid (BQA) and is further polymerized extracellularly toward pyomelanin. Pyomelanin possesses a high degree of structural complexity and heterogeneity similar to the other types of melanin, pheomelanin, and eumelanin. However, in natural processes, pyomelanin is produced under certain pathological conditions, leading to variations of the chemical subunits and specific structures of pyomelanin depending on its source and formation conditions. This structural heterogeneity allows for a broader range of energy absorption and dispersion enabling pyomelanin to effectively shield against various forms of radiation, including ultraviolet (UV), visible light, ultraviolet (UV), and even ionizing radiation. Additionally, due to its heterogenic chemical structure, pyomelanin exhibits an extended conjugated π-electron system within its polymeric structure. This extended conjugation, resulting from the arrangement of its building blocks, enables pyomelanin to efficiently absorb, and disperse electromagnetic radiation. The extensive delocalization of electrons facilitates the absorption of a broader spectrum of radiation, thereby enhancing pyomelanin's radiation shielding capabilities compared with eumelanin and pheomelanin.

**Figure 1:**
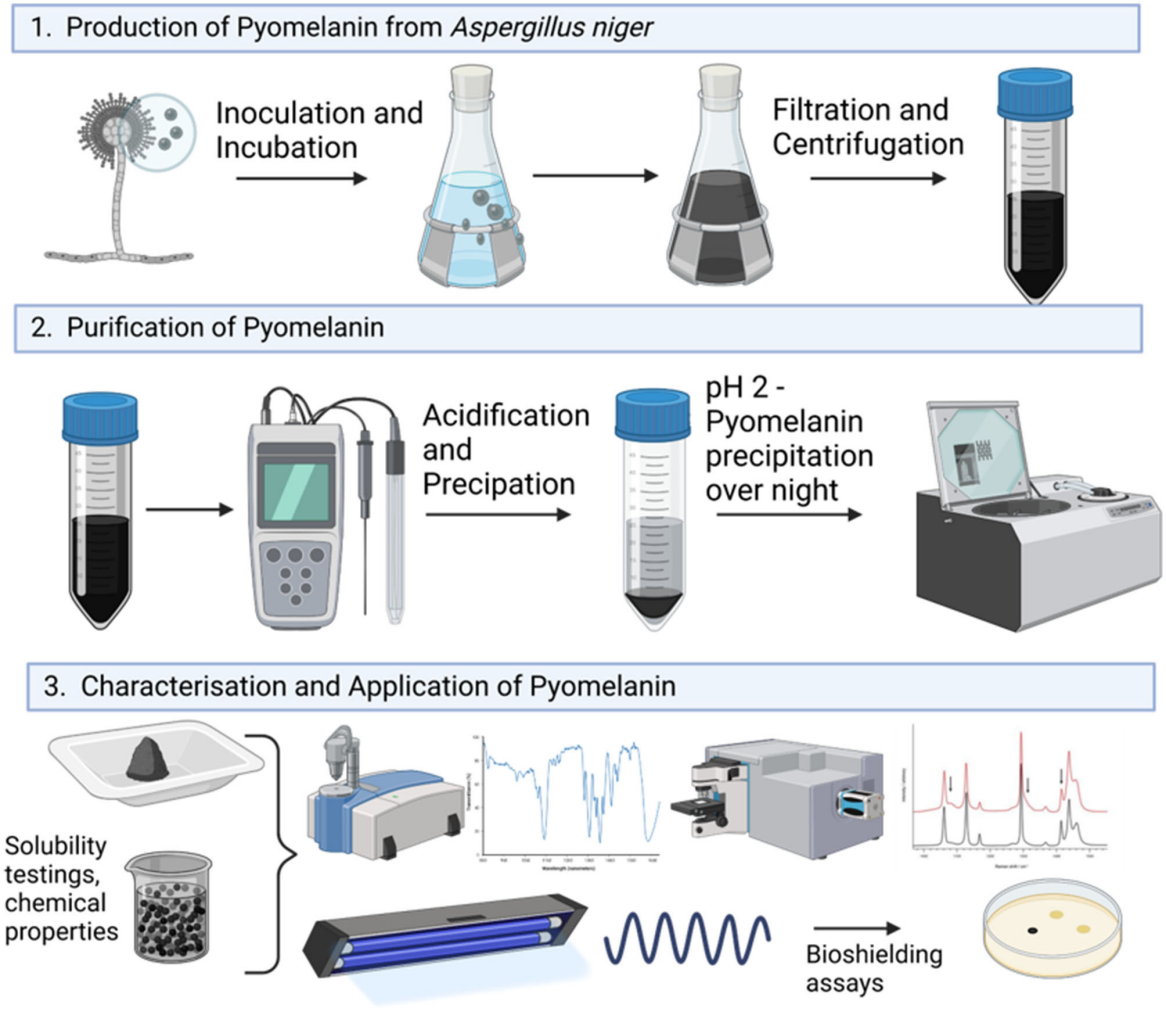
Graphical overview of the experimental workflow of this study.

To date, the genetic basis and biochemical route for the DHN-melanin biosynthetic route have only partly been described for *A. niger* (Jørgensen et al., 2011). Furthermore, as the deletion of the *fwnA* gene encoding the initial polyketide synthase reaction did not result in colorless spores (they are still fawn), the observed remaining coloration does suggest that other compounds of primary or secondary metabolism might contribute to a colored appearance of the colonies (Jørgensen et al., 2011). In this study, we provide genetic and metabolic evidence that *A. niger* is indeed capable of naturally producing and secreting pyomelanin on a g/L scale and additionally demonstrate that this fungal pyomelanin can be successfully applied as a radiation shield (Figure 1). Especially due to the fact that the generated pyomelanin is synthesized extracellularly, unlike other known fungal melanins (L-DOPA- or DHN-melanin) which are deposited within the cell wall, the need for cell lysis is eliminated and reduces associated costs during purification, which makes it a valuable resource for biotechnology, biomedicine, and space industry. In future, it could potentially be utilized as a multi-purpose melanin-based biopolymer and material coating for bio-shielding on crewed space missions.

## 2. Materials and methods

### 2.1. Strains, media, and culture conditions

*A. niger* strains used in this study are summarized in Table 1. Spore suspensions were prepared from 3 days (for N402 and MA93.1) colonies grown on complete media (CM) agar [55 mM glucose, 11 mM KH_2_PO_4_, 7 mM KCl, 178 nM H_3_BO_3_, 2 mM MgSO_4_, 76 nM ZnSO4, 70 mM NaNO_3_, 6.2 nM Na_2_MoO_4_, 18 nM FeSO_4_, 7.1 nM CoCl_2_, 6.4 nM CuSO_4_, 25 nM MnCl_2_, 174 nM EDTA, 15 g/L of agar supplemented with 0.5 % (w/v) yeast extract, and 0.1 % (w/v) casamino acids]. The spores were gently harvested with a sterile cotton swab and suspended in 0.9 % sodium chloride (NaCl). The resulting spore suspensions were filtered through sterile miracloth to remove hyphal fragments. Fresh spore suspensions, <2 weeks old, were used for all inoculations. Liquid shake flask cultivations were performed in CM medium for 6 days at 30°C at 200 rpm, after inoculation with 1 mL of 10^6^ sps/mL in 200 mL flasks.

**Table 1:** *A. niger* strains used in this study.

**Strain**	**Relevant genotype**	**Description**	**References**
N402		Wild type	(Bos et al., 1988)
MA93.1	*ΔfwnA*	N402 derivative in which the putative polyketide synthase FwnA has been deleted. Spores produce a fawn pigment.	(Jørgensen et al., 2011)
MF41.3	*kusA^−^, hisB^−^, pyrG^−^*	N402 derivative in which the *kusA* gene has been disrupted to improve homologous recombination frequency. The strain is both histidine and pyrimidine auxotroph which can be harnessed for genetic transformation.	(Fiedler et al., 2017)
OS3.1	*ΔhppD, pyrG^−^*	MF41.3 derivative in which the *hppD* gene (An11g02200) has been deleted using *hisB* as an auxotrophic marker. The strain is still pyrimidine auxotroph.	This study
OS4.3	*ΔhmgA, pyrG^−^*	MF41.3 derivative in which the *hmgA* gene (An11g02180) has been deleted using *hisB* as an auxotrophic marker. The strain is still pyrimidine auxotroph.	This study

### 2.2. Molecular techniques

All molecular techniques were performed according to standard procedures (Green and Sambrook, 2012). Protoplast-mediated transformation of *A. niger*, genomic DNA extraction, diagnostic PCR, and Southern blot were performed as described earlier (Fiedler et al., 2017). Quantitative PCR was performed according to Polli et al. (2016) using a 10 μl reaction with 2x Blue SYBR Green Mastermix (Biozyme) on an AriaMX Real-time PCR System (Agilent Technologies). *A. niger* strains MF41.3 and N402 were used as negative and positive controls, respectively.

### 2.3. Generation of *hppD* and *hmgA* mutant strains

All plasmids used in this study were constructed using DNA parts available in the Fungal MoClo Toolkit (Addgene Kit # 1000000191; Mózsik et al., 2021). A measure of 0.5 μl of the required type II restriction enzyme (BpiI, BsaI, or Esp3I, Thermo Scientific) was used per 15 μl reaction and 1 μl of T4 DNA ligase (Thermo Scientific). Golden Gate reactions were performed for 25 cycles (5 min at 37°C, 5 min at 16°C) and a final inactivation step of 10 min at 70°C to denature enzymes. A measure of 7.5 μl of the reaction mixture was transformed into 50 μl of TOP10 chemically competent *Escherichia* coli cells (NEB) using a heat shock at 42°C for 30 s. Two protospacers for Cas9 within the coding region of An11g02180 (*hmgA*) and An11g02200 (*hppD*) were identified using CCTop (Stemmer et al., 2015), and the corresponding sgRNA expression plasmids were constructed using pFTK086 to express the sgRNA under control of the tRNA promoter An08e08800. Sanger sequencing (LGC Genomics) was performed to validate the correct insertion of the protospacer into the sgRNA cassette. For the preparation of donor DNA, KAPA HiFi Polymerase was used (Roche). All primers used in this study are listed in Supplementary material 1. Protoplasts of strain MF41.3 were transformed using a CRISPR approach that harnessed Cas9-aided short-flank (75 bp) donor DNA for integration (Pohl et al., 2016).

### 2.4. Pyomelanin production, purification, and quantification

For pyomelanin production, 200 mL of CM was supplemented with 1 mM uridine and 2.2 mM L-tyrosine (hereafter abbreviated CM + Uri + Tyr.) in a 500-mL Erlenmeyer flask with 1 × 10^8^ spores/mL of strain OS4.3 and incubated for 6 days at 150 rpm and 30°C. After cultivation, the cultural broth was centrifuged at 4,500 rpm for 20 min. This process was repeated three times. After centrifugation, the collected supernatant was filtered through a sterile miracloth filter to remove any leftover hyphal fragments and was kept in Falcon tubes for further investigation. For the purification of the Pyo_Fun_, a modified protocol by Schmaler-Ripcke et al. (2009) was followed. In brief, the supernatant containing pyomelanin was adjusted to pH 2 using 1 M HCl. The samples were precipitated at room temperature in the dark for 15 h. After precipitation, the tubes were centrifuged at 4,500 rpm for 20 min, the supernatant was discarded, and the Pyo_Fun_ pellet was saved for lyophilization in an Eppendorf Concentrator plus complete system with an integrated diaphragm vacuum pump with rotor F-45-48-11, 230V/50–60Hz. Before lyophilization, the pellet was rinsed with ddH_2_O three times, and during washing, several melanin pellets can be concentrated into the same tube(s) which lowers the general time needed for lyophilization. Aliquots of Pyo_Fun_ were placed lyophilized and concentrated using the V-Aq program for 9 h at 4°C. The full process was performed 10 times (*n* = 10), and the yield was determined by weighing the lyophilized Pyo_Fun_ using a precision scale (Sartorius) which was referred to as the cultivation volume (200 mL). Additionally, an *in vitro* synthesized pyomelanin (Pyo_Syn_) was used as the control for all the following experiments. The synthetic pyomelanin was produced in accordance with the protocol described by Schmaler-Ripcke et al. (2009). In brief, 10 mM HGA (Sigma Aldrich) autoxidizes at pH 10 (adjusted with 1M NaOH) under constant stirring for 3 days in the dark at room temperature (RT). The lyophilization and quantification were carried out in the same way as described for Pyo_Fun_.

### 2.5. Fourier-transform infrared spectroscopy

FTIR was used to characterize both lyophilized fungal and synthetic pyomelanin using a Spectrum One^TM^ from Perkin-Elmer Inv. #429 with the following specifications: Analysis mode: attenuated total reflection (ATR) on diamond crystal and Measuring range: wavenumber 4,000 to 650 cm^−1^. For FTIR measurements (*n* = 3 for every measurement), approximately 1 mg of fungal pyomelanin (Pyo_Fun_), synthetic pyomelanin (Pyo_Syn_), and corresponding precursors for pyomelanin production, L-tyrosine (Sigma Aldrich), and homogentisic acid (Sigma Aldrich) were transferred to the sample plate and pressed onto the ATR diamond window using a stamp.

### 2.6. Raman analysis

Raman spectra for Pyo_Fun_, Pyo_Syn_, L-tyrosine, and homogentisic acid were determined. For the spectral acquisition, a small amount of respective powder was transferred onto the CaF2 slide, and spectra were acquired directly. All the samples were analyzed using the commercial Renishaw Raman spectrometer (Renishaw inVia Raman Spectrometer, Renishaw plc., Wotton-under-Edge, UK) with a 785-nm single-mode diode laser as an excitation source. The laser beam was focused onto a sample using a microscope objective (Leica, Wetzlar, Germany, N PLAN EPI, magnification 50 × , working distance 0.5 mm, and numerical aperture 0.75). The dimensions of a laser spot shape typical for the Renishaw inVia instrument were ~2 μm × 10 μm, with a full axial depth of the excitation region at 8 μm (Mlynáriková et al., 2015; Rebrošová et al., 2017). Before each spectral acquisition, the laser beam was refocused onto the individual fungal spore or the powder to stay within the focal depth of the laser beam excitation and the imaging optics. Considering the possible variability of individual fungal spores, 10 measurements were taken per strain, each for a different spore. Similarly, 10 measurements were taken per powder sample. Five-second acquisition time and 100% laser power (~140 mW on a sample) were used for all powders. Due to a significant autofluorescence of fungal spores in the range 614–1,724 cm^−1^, the acquisition settings had to be adjusted. To acquire each individual spectrum, accumulations of 10 × 1 s and 10% laser power were used (~14 mW).

The acquired Raman spectra were analyzed using an in-house written program based on MATLAB software (MathWorks, Natick, MA, USA). First, high-frequency noise was removed using Savitzky–Golay filtering (4th order, width 15 points). Then, the spectra were treated with rolling circle filtering (50 passes, 500 points circle radius) to suppress a fluorescence background. Finally, the area of spectra was normalized to 1, and a threshold of 0.001 was applied. A comparison of spectra from individual fungal spores belonging to different *A. niger* strains was made using centered principal component analysis (PCA). The groups were marked by ellipsoids with a Mahalanobis distance of 2.15, corresponding to the 90% confidence interval (De Maesschalck et al., 2000; Rebrošová et al., 2017).

### 2.7. Physicochemical property analysis

To characterize the solubility properties of Pyo_Fun_ and Pyo_Syn_, 0.1–0.5 mg/mL of the respective lyophilized pyomelanin was dissolved in 1 mL of EtOH (100%), 0.1 M KOH, DMSO (100%), and ddH_2_O and vortexed for 10 s each. Solubility was assessed visually. Additionally, to test for decolorization with oxidizing agents, 100 μL of 30% H_2_O_2_ was added to 900 μL of dissolved Pyo_Fun_, and oxidation was stopped after 10 min or 20 min using catalase (0.1 mg/mL, from bovine liver, Sigma Aldrich). The bleaching process was then evaluated via UV-Vis spectroscopy using a multidetection microplate reader (Infinite M200 PRO, Tecan).

### 2.8. Antioxidant assay

A 2,2,1-diphenyl-1-picrylhydrazyl (DPPH) radical scavenging assay described earlier (Chen et al., 2013) was used with some modifications. In brief, DPPH is a free radical with a dark violet color that provides hydrogen acceptor capability to antioxidants. Its absorption maximum occurs at 515 nm. When antioxidants react to DPPH by providing an electron or hydrogen atom, the free radical DPPH is reduced to 2,2-diphenyl-1-hydrazine (DPPH-H), and its dark violet color changes toward a colorless or pale-yellow/orange color, which can be detected using a multidetection microplate reader (Infinite M200 PRO, Tecan).

To evaluate the antioxidant activities of Pyo_Fun_, a DPPH stock solution was prepared by mixing DPPH free radical (C_18_H_12_N_5_O_6_, Sigma Aldrich) with 100% EtOH to create a stock solution (0.002%) and left to incubate for 2h at 4°C. This stock solution was then vigorously mixed with each sample and incubated in the dark at room temperature. Two comparative samples were taken along, one with known high reactive oxygen species (ROS) scavenging activity (ascorbic acid 5 mg/mL of stock solution, diluted 1:50) and one with low ROS scavenging activity (DMSO 100%, diluted 1:50). The two samples of Pyo_Fun_ (pure culture supernatant, diluted 1:50 and purified pyomelanin powder dissolved in H_2_O, 5 mg/mL, diluted 1:50) and one negative control (the solvent DMSO and DPPH) were measured. The discoloration was measured at 517 nm for 30, 40, and 60 min, respectively, after the reaction was initiated. All tests were performed in technical triplicates (*n* = 3). The percentage of antioxidative effect was calculated using the following equation:


$$\begin{array}{l} Scavenging\;activity\;\left(\%\right)\\ =\;{\left(\frac{Absorbance\;Control-Absorbance\;Sample}{Absorbance\;Control}\right)}^{*}100 \end{array}$$

### 2.9. Radiation shielding assay

To characterize the physical and biological shielding properties of Pyo_Fun_ and Pyo_Syn_, 0.5 mg/mL of lyophilized pyomelanin was dissolved in 1 mL of 0.1 M KOH under sterile conditions and pipetted in quartz glass cuvettes (QS 0.5, Hellma). A measure of 1 mL of 0.1 M KOH was additionally pipetted in a cuvette to be used as a negative control (solvent blank). These quartz glass cuvettes are used as “melanin filters” and contain a homogenous mixture of solved pigment. As UV-C radiation can transmit through the quartz glass cuvettes, one can evaluate only the shielding capacities of our dissolved pigments. To evaluate the physical shielding of UV-C radiation, the cuvettes were placed individually on top of the dosimetry sensor of a UVX Radiometer (Analytic Jena) to shield the monochromatic wavelength of 254 nm originating from UV-Lamp (VL-215-LC, Vilber Lourmat, SN.: 14 100595). The corresponding dose rate reaching the dosimetry sensor was recorded. This was performed six times for each cuvette filled with pyomelanin or the solvent control for each of the two radiation forms (see Supplementary Figure SI 4).

Fungal spores of the Δ*fwnA* were tested against the UV-C dose that is required to eliminate 90% of the wild-type spores (LD_90_; 1,038 J/m^2^, Cortesão et al., 2020b). Spores of *A. niger* were exposed to UV-C (254 nm) in a 96-well plate with an initial concentration of 10^6^ spores/ml in 200 μL of saline solution (0.9% (w/v) NaCl). The concentration of 10^6^ spores/ml ensures a spore monolayer and prevents survival due to shielding by the spores themselves. The radiation dose was adjusted through exposure time since the intensity (distance to object) of the UV lamp was kept constant. UV-C exposure time was calculated using the following formula (Cortesão et al., 2020b):


$$\begin{array}{l} t\left(s\right)=\;\frac{R\;\left(J/m\right)\;x\;100}{d\;\left(\mu W/cm2\right)} \end{array}$$

where *t* = time (in seconds), *R* = desired radiation dose (in J/m^2^), and *d* = dosimeter value for UV fluence (in μW/cm^2^).

After irradiation, 30 μL of the sample were taken in triplicates from the spore suspension within each well, and viability was calculated by the ability to form colonies (colony forming units, CFU). Radiation exposure included at least three biological replicates per strain and was performed two independent times (*n* = 6). Samples were serially diluted up to 10^−8^ using a 96-well plate, with each well having a total volume of 300 μl. To count the CFUs, 20 μl of each dilution was plated out in triplicate on one-eighth of a Petri dish with MM agar which contained Triton X-100 (0.05%) to facilitate counting. The plates were incubated for 2 days at 30°C before the colonies were counted.

The survival of fungal spores after irradiation was additionally tested using an oCelloScope™ (BioSense Solutions ApS, Farum, Denmark) at 22°C for 48 h. The oCelloScope™ is a digital time-lapse microscopy technology that generates high-resolution micrographs of microorganisms while growing. This is achieved by scanning through a growth chamber, creating a series of images on a z-axis. The oCelloScope™ quantifies fungal mass per well on the best focus level of the z-axis images per timepoint by quantifying the number of pixels belonging to the fungus and to the background (Winters et al., 2022). Germination of fungal spores and formation of hyphae were micrographed in 30 min intervals after samples were diluted to 2.3 × 10^5^ spores/mL and allowed to rest for 10 min before image acquisition. The oCelloScope™ then analyzed corresponding changing fungal mass over time per well (*n* = 6) using the build-in SESA fungi algorithm (Aunsbjerg et al., 2015).

## 3. Results

### 3.1. Genetic basis of pyomelanin formation in *A. niger*

**Figure 2:**
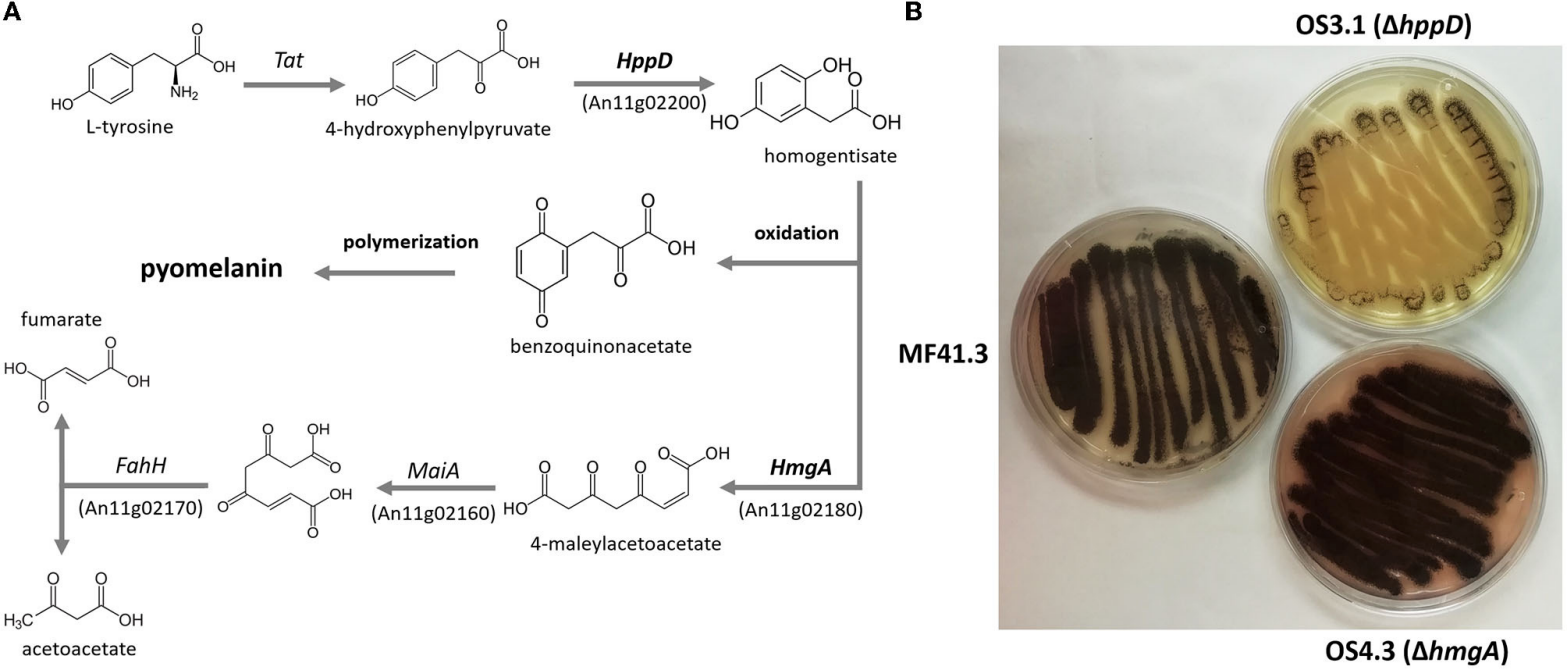
**(A)** Pathway for pyomelanin formation in *A. fumigatus* after (Heinekamp et al., 2013). The orthologous genes in *A. niger* were predicted using MultiGeneBlast. For homology on the protein level, please see Supplementary material SI 1. **(B)** Growth phenotypes of *A. niger* strains with the knockout of *hppD* (strain OS3.1) and *hmgA* (strain OS4.3) compared with the parental strain MF413 grown on minimal medium agar plates supplemented with 2mM L-tyrosine.

As several biosynthetic routes are followed in fungi to produce L-DOPA melanin (= eumelanin), DHN-melanin (allomelanin), or pyomelanin, we used orthology screening to identify *A. niger* genes that encode enzymes predicted to be involved in the degradation of L-phenylalanine/L-tyrosine and thus pyomelanin formation. We focused on the well-described pyomelanin pathway in *A. fumigatus* (Schmaler-Ripcke et al., 2009) and could indeed identify gene candidates based on MultiGeneBlast analyses (Blin et al., 2013). The pathway involves six genes, which physically colocalize within a gene cluster. All pairwise gene identities between *A. fumigatus* and *A. niger* are given in Figure 2A, Supplementary Tables SI 1, SI 2, and Supplementary Figures SI 1, SI 2. We decided to delete two central genes of this pathway in *A. niger*–*hppD*, which catalyzes the precursor of pyomelanin, homogentisic acid (HGA), and *hmgA*, which degrades HGA using CRISPR-Cas9 technology (see Materials and methods section). We received several *A. niger* transformants for each deletion approach and verified successful deletion of *hppD* and *hmgA*, respectively, via diagnostic and quantitative PCR (Supplementary material SI 3). A comparative phenotypic analysis clearly demonstrated that the deletion of *hppD* resulted in strains that nearly lost the ability to produce any dark pigment (e.g., OS3.1) when cultured in the presence of L-tyrosine, whereas the deletion of the *hmgA* gene resulted in strains that secreted much more of a dark brown pigment, supposedly pyomelanin, into the medium agar (e.g., strain OS4.3).

### 3.2. Production and verification of pyomelanin from *A. niger*

**Figure 3:**
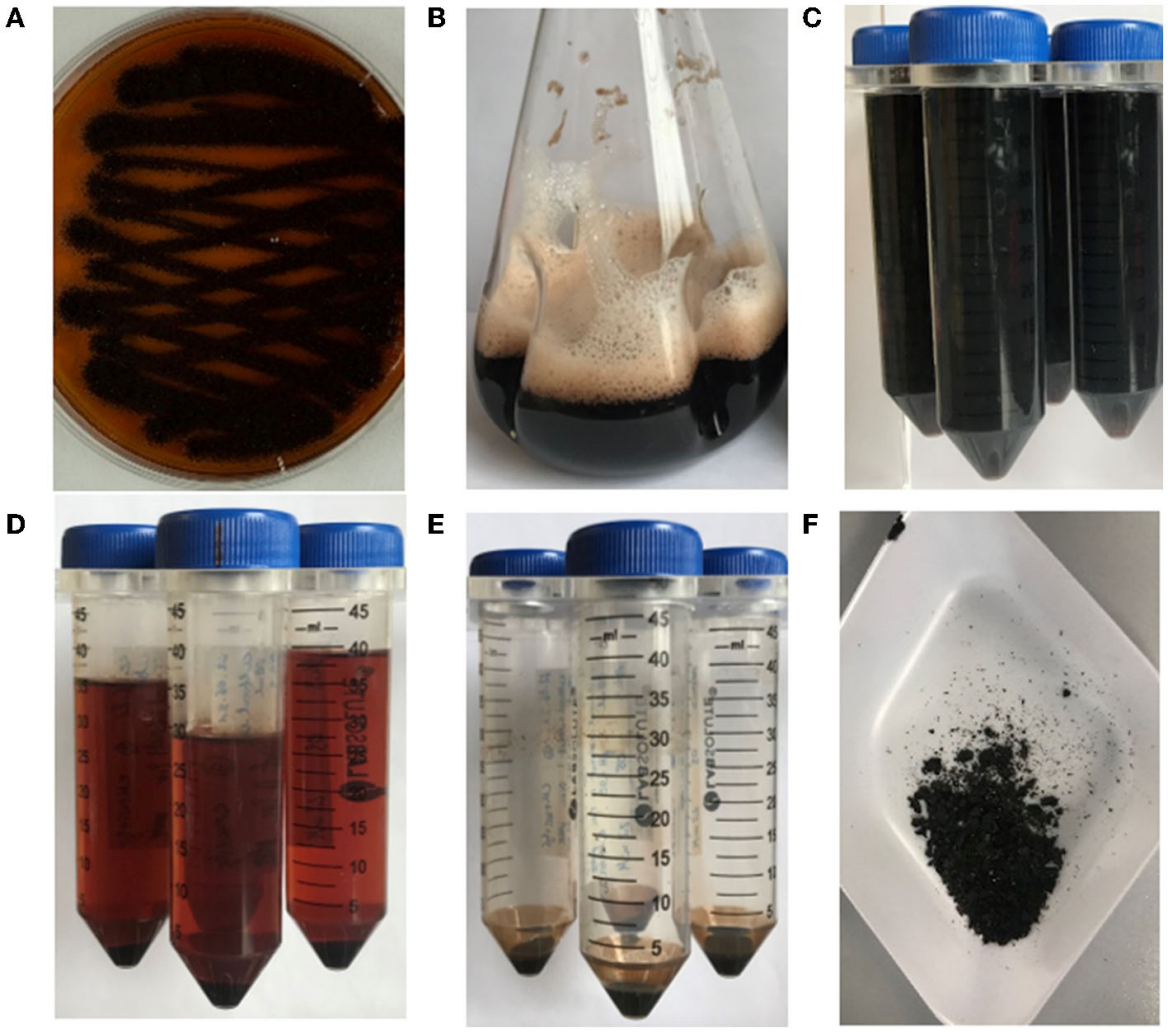
Production and purification of Pyo_Fun_ from *A. niger* strain OS4.3. **(A)** Strain OS4.3 incubated for 4 days on a CM agar supplemented with 2.2 mM L-tyrosine. **(B)** Strain OS4.3 cultivated in liquid CM supplemented with 2.2 mM L-tyrosine for 6 days. **(C–F)** Acidification and a final lyophilization step led to pure Pyo_Fun_.

To confirm that the dark brown pigment secreted by strain OS4.3 is indeed pyomelanin (hereafter abbreviated as Pyo_Fun_), we first established a pyomelanin production and purification protocol for submerged *A. niger* cultures based on the study published earlier for *A. fumigatus* (Perez-Cuesta et al., 2020). As shown in Figure 2 and described in detail in the Materials and Methods section, the production of substantial amounts of Pyo_Fun_ can be induced in strain OS4.3 by adding L-tyrosine to the culture medium. When strain OS4.3 was cultivated for 6 days in liquid CM medium (Figures 3A–C), we could isolate approximately 1.5 g Pyo_Fun_ out of 1 L of culture supernatant. Remarkably, Pyo_Fun_ seems to be mostly present extracellularly, from where we were able to easily purify it via acidification (pH 2) (Figures 3D, E) and lyophilization into a powder that appeared dark brown to black in color (Figure 3F).

In order to verify that the dark brown/black powder is indeed Pyo_Fun_, it was subjected to different spectroscopic analyses including UV-Vis, FTIR, and Raman spectroscopy. In this study, a synthetically produced pyomelanin (Pyo_Syn_) was taken along, which was proven earlier to show the same spectral characteristics as fungal pyomelanin (Lorquin et al., 2021). Pyo_Syn_ is thus an excellent positive control and can be easily obtained through the autooxidation of HGA when subjected to alkaline conditions (see Materials and Methods section).

**Figure 4:**
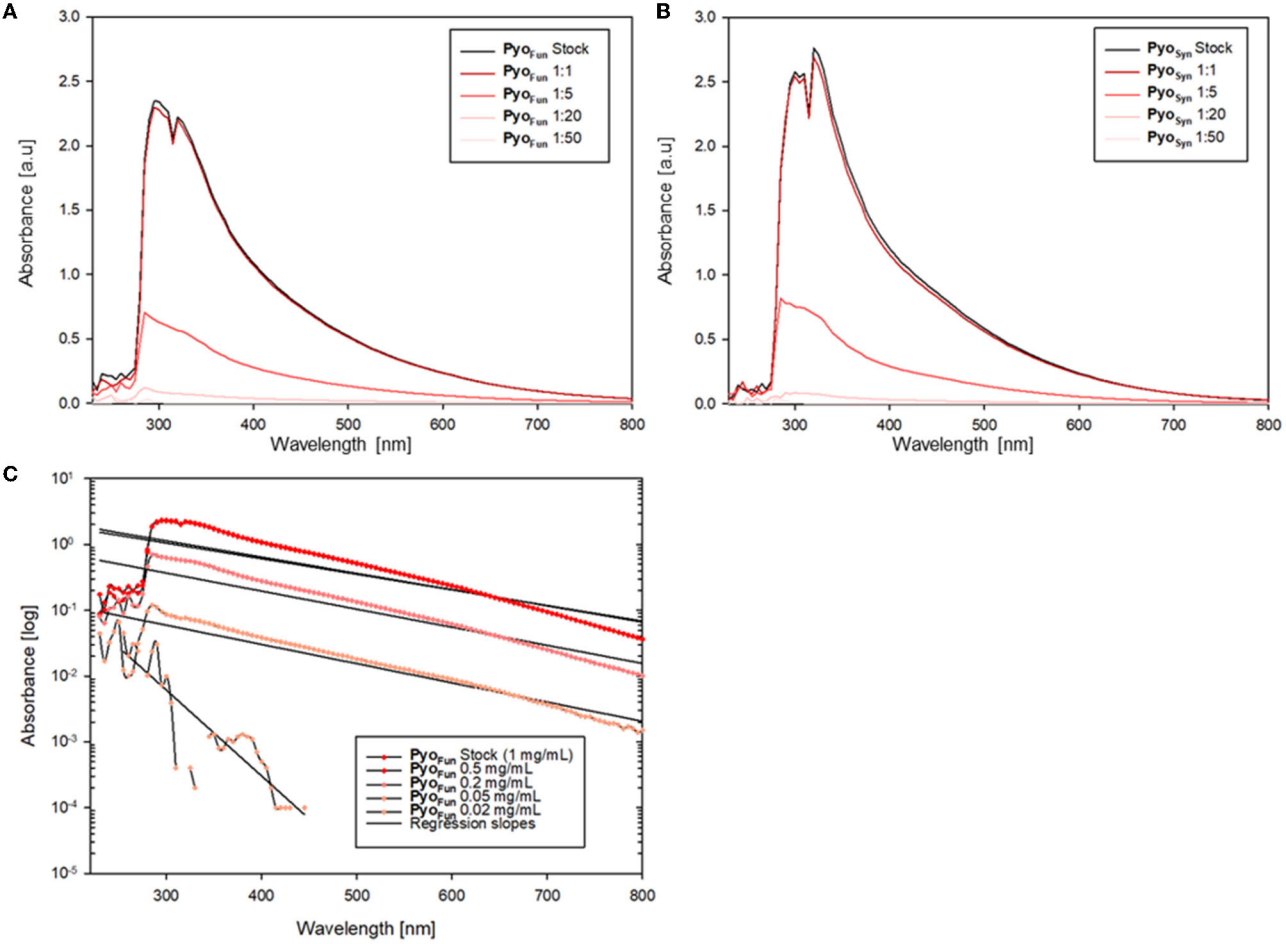
UV-Vis spectra of Pyo_Fun_ and Pyo_Syn_. Spectra for Pyo_Syn_ **(A)** and Pyo_Fun_ **(B)** when measured in different concentrations/dilutions as indicated. **(C)** Spectrum of Pyo_Fun_ plotted exemplarily as a logarithmic function and shows the regression as linear negative plots, indicating the constant decrease in the absorbance spectra of Pyo_Fun_ in higher wavelengths.

As shown in Figures 4A, B, both Pyo_Fun_ and Pyo_Syn_ exhibit similar UV-Vis spectra and have their absorbance maximum between 280 and 335 nm, which is characteristic for most types of melanins (Pralea et al., 2019). We used different concentrations of both compounds and detected an exponential increase of absorption in wavelength under 500 nm and a gradual decrease of absorbance in higher wavelengths for both, Pyo_Fun_ and Pyo_Syn_. The maximum absorbance peak of Pyo_Fun_ (2.4 [a.u]) is slightly lower than that of Pyo_Syn_ (2.6 [a.u.]). In both trajectories, the two highest concentrations (1 mg/mL and 0.5 mg/mL) depicted a double peak between 280 and 335 nm. Such behavior can be expressed mathematically when plotting the logarithms of each absorbance curve against the wavelength. The absorption regression lines of the original curves are linear with negative slopes (Figure 4C), which agrees with melanin data from the literature (Lorquin et al., 2021).

**Figure 5:**
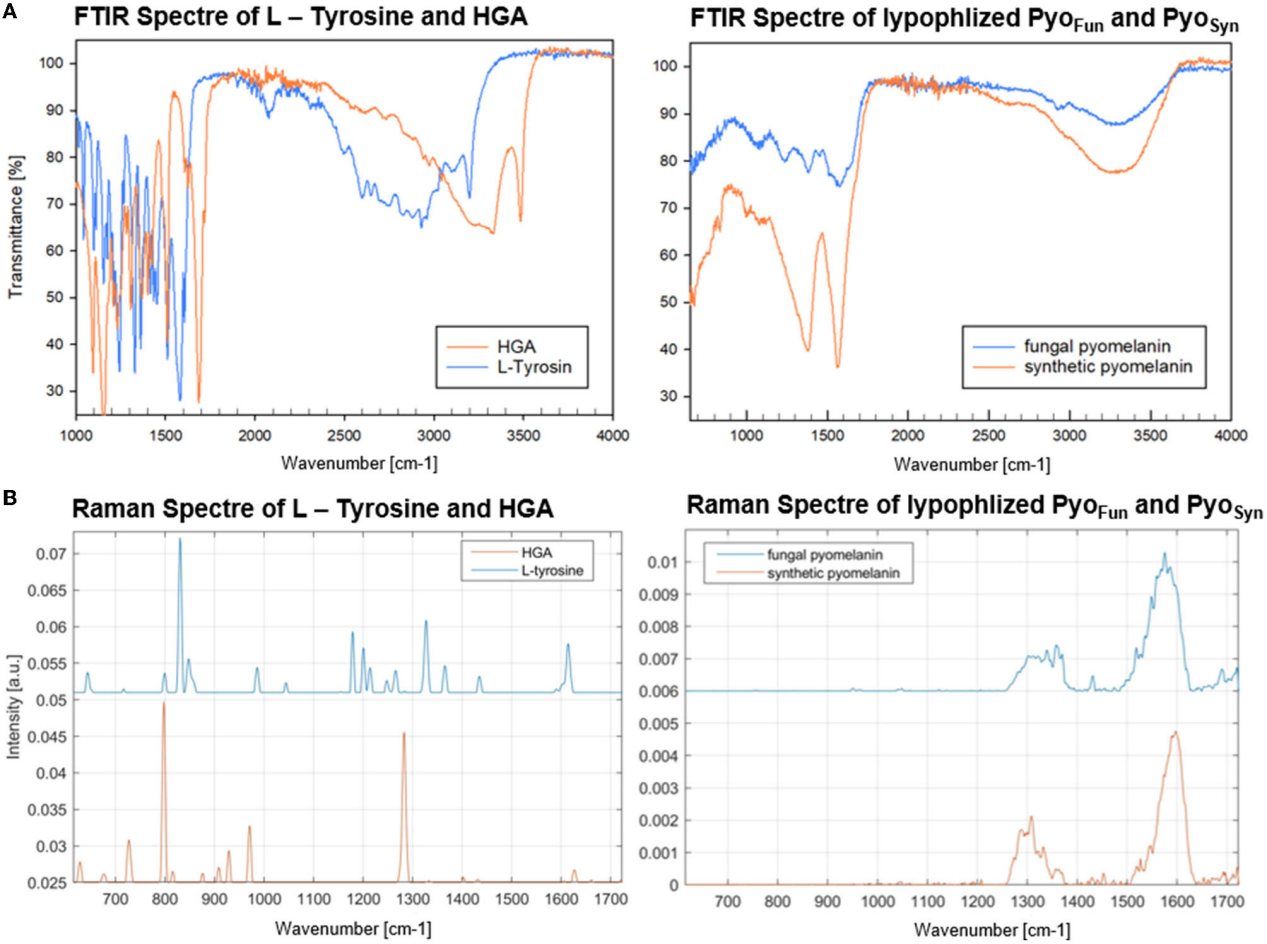
FTIR **(A)** and Raman spectra **(B)** of Pyo_Fun_, Pyo_Syn_, L-tyrosine, and HGA. For details, see Materials and methods section.

Data from FTIR and Raman spectroscopy also confirmed that both Pyo_Fun_ and Pyo_Syn_ are very similar compounds and display spectra which are characteristic of melanins (Figure 5). For these analyses, we took the precursors L-tyrosine and HGA along. FTIR frequencies of Pyo_Fun_ and Pyo_Syn_ show melanin characteristic bands and transmittance peaks covering regions between 3,600–2,900 cm^−1^, 1,600–1,500 cm^−1^, and 1,400–1,300 cm^−1^ (Figure 5A; note that the Pyo_Syn_ FTIR spectra are better resolved). The corresponding assignment of the prominent bands most likely indicates symmetric carboxylate stretching vibrations (COO–) (1,580 cm^−1^) and polymeric O-H groups (3,400 cm^−1^). Within the fingerprint region (400–1,400 cm^−1^), there is one distinct peak at 1,390 cm^−1^ for Pyo_Syn_ which could indicate a C-H bending. All peaks within the transmittance curve seen in Pyo_Syn_ are more intense than for Pyo_Fun_ but they show a similar trajectory pattern. The prominent peaks in the Pyo_Syn_ spectrum (at 1,375, 1,575, and 3,287 cm^−1^) are generally approximately 30% lower in transmittance (%) than Pyo_Fun_. In comparison to the pyomelanin samples, the FTIR transmittance spectra of the two precursors L-tyrosine and HGA show clear differences toward each other and to the pigments. They exhibit very sharp bands between regions 1,000 cm^−1^ indicating a different molecular structure. HGA additionally shows defined peaks at approximately 3,333.4 cm^−1^ and 3,479.2 cm^−1^ and L-tyrosine at 3,195.4 cm^−1^. The Raman spectra shown in Figure 5B provide complementary information. The relative intensity of the all Raman bands in both the precursors differs from the bands visible in both pyomelanin. HGA and L-tyrosine exhibit high-frequency vibrations between 800 and 850 cm^−1^. HGA shows another prominent band at 1,290–1,300 cm^−1^ in contrast to L-tyrosine which exhibits several medium strong vibrations between 1,190 and 1,350 cm^−1^. Significant similarities can be observed in the vibration frequencies of Pyo_Fun_ and Pyo_Syn_. Both pigments show medium strong bands between 1,290 and 1,390 cm^−1^ (stretching of the C-C bond) and a higher frequency vibration between 1,550 and 1,621 cm^−1^, which is indicative of aromatic or heterocyclic ring systems.

### 3.3. Physicochemical characterization of pyomelanin from *A. niger*

**Figure 6:**
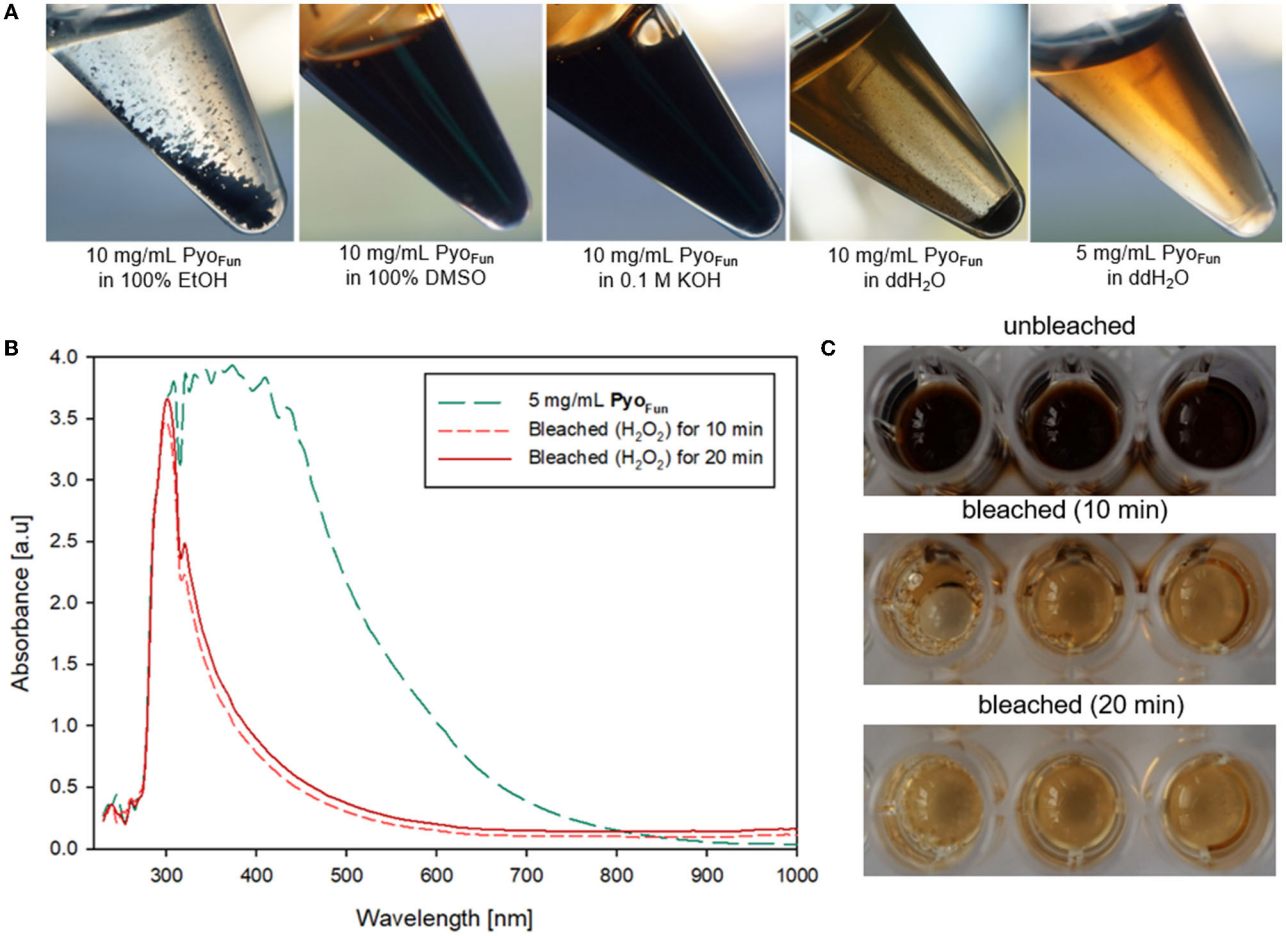
Physicochemical characterization of Pyo_Fun_. **(A)** Solubility tests for two concentrations of Pyo_Fun_ in different solvents as indicated. Note the solubility limit in water at higher pigment concentrations (>5 mg/mL). **(B)** UV-Vis spectra of Pyo_Fun_ after bleaching 5 mg/ml of Pyo_Fun_ with 30% H_2_O_2_. **(C)** Pyo_Fun_ loses its dark colorization through bleaching.

The different spectral analyses demonstrated that Pyo_Fun_ and Pyo_syn_ are identical molecules and display spectral characteristics of melanins published in the literature. We next analyzed physicochemical properties of Pyo_Fun_ with a focus on solubility and reactivity since different kinds of melanin display distinctive solubility and reactivity properties (Pralea et al., 2019). We tested the solubility of Pyo_Fun_ in water (aqueous solvent), ethanol (100 % EtOH, polar solvent), dimethylsulfoxide (100 % DMSO, polar aprotic solvent), and potassium hydroxide (0.1 M KOH, aqueous alkaline solvent). Figure 6A shows that 10 mg/mL of purified and lyophilized Pyo_Fun_ is poorly soluble in water, well soluble in dimethylsulfoxide and potassium hydroxide, and insoluble in ethanol.

Melanins are known to be bleachable when treated with different oxidizing agents including hydrogen peroxide (Pralea et al., 2019). Bleaching is thus a common method to qualitatively determine any antioxidant activity of a compound of interest. We treated Pyo_Fun_ with 30 % H_2_O_2_ solution and stopped the reaction after 10 and 20 min, respectively, via the addition of 0.1 mg/mL of catalase. UV-Vis spectral analyses showed the loss of absorbance capacity of Pyo_Fun_ between 300 and 500 nm (Figure 6B), which parallels the loss of dark colorization. Indeed, the bleaching result was already visually assessable by eyes (Figure 6C), demonstrating that Pyo_Fun_ exerts antioxidant activities.

**Figure 7:**
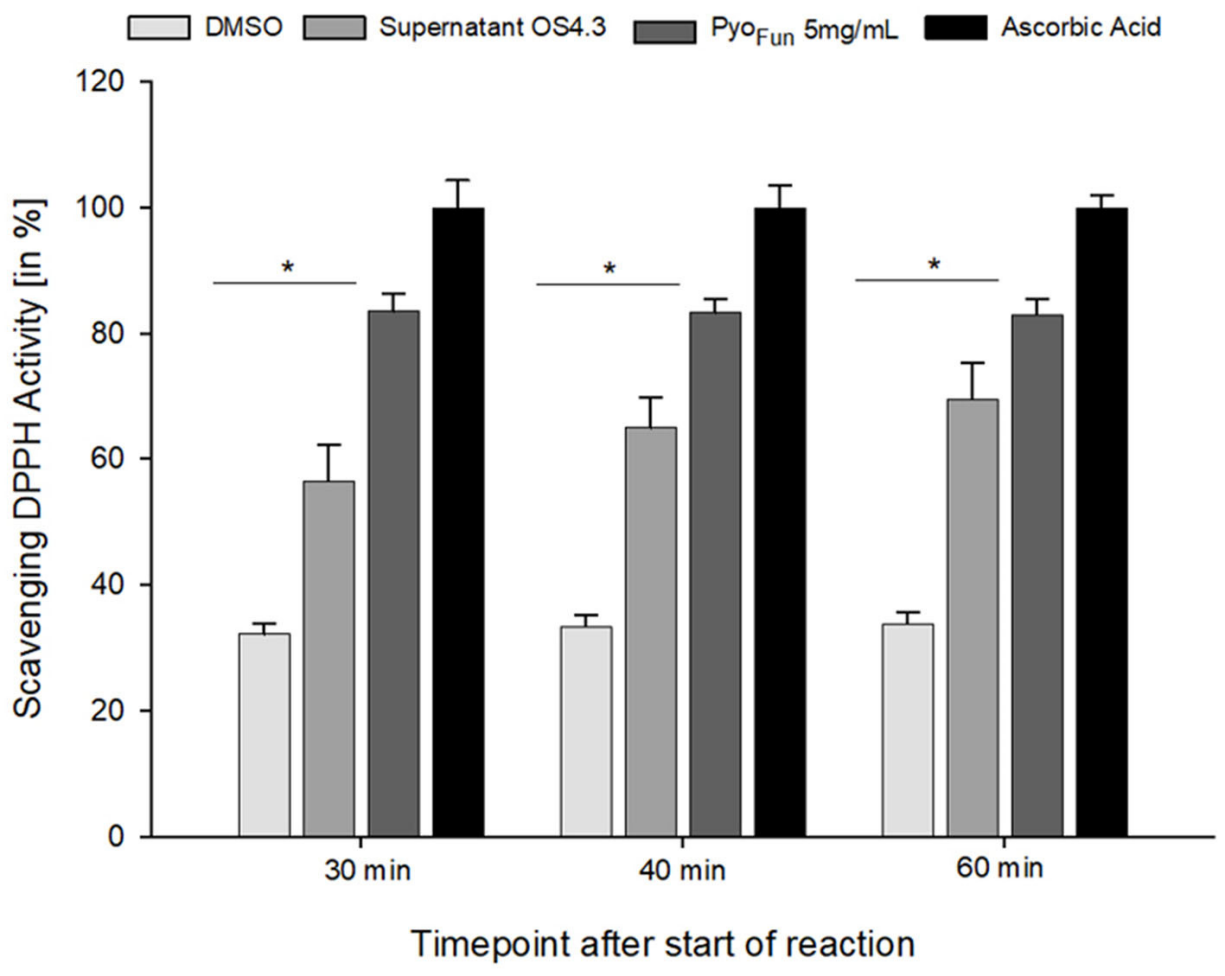
ROS scavenging activity of Pyo_Fun_. A DPPH radical scavenging assay was performed in triplicates for four samples: filtered culture supernatant from strain OS4.3, lyophilized Pyo_Fun_ (5 mg/mL), ascorbic acid (5 mg/mL, positive control), and DMSO (100%, negative control). For all time points measured, the statistical significance of the two groups tested (Welch's *t*-test, 95% confidence interval) was determined: *p* = 0.019 for Pyo_Fun_ vs. DMSO and *p* = 0.04 for Pyo_Fun_ vs. culture supernatant of strain OS4.3, respectively. Standard deviation is given.

To quantitatively assess the antioxidant capacity of Pyo_Fun_, we performed a ROS scavenging assay with culture supernatant from strain OS4.3, lyophilized Pyo_Fun_ pigment (5 mg/mL), ascorbic acid (positive control), and DMSO (negative control; see Materials and Methods section for details), and measured ROS scavenging after 30, 40, and 60 min, respectively. Figure 7 illustrates that the ROS scavenging capacity of lyophilized Pyo_Fun_ was found to be approximately 80% of the capacity of ascorbic acid. Moreover, the unconcentrated culture supernatant of strain OS4.3 obtained through filtration also showed approximately 60% of the radical scavenging capacity of ascorbic acid.

### 3.4. Radioprotection capacity of pyomelanin from *A. niger*

The higher order structures of the different kinds of melanin form the basis for their broad optical absorption and potent antioxidant capacities but also for their ability to protect against ionizing radiation such as UV and X-ray (Cordero and Casadevall, 2017). Radioprotection by melanins is thought to be mediated by three main mechanisms: (i) Melanins are able to absorb radiation energy while dissipating the energy through heat. (ii) Melanins trap and neutralize free radicals and ROS which become released by cellular molecules (DNA, proteins, and lipids) as a consequence of radiation (biological shielding; Esbelin et al., 2013; Cordero and Casadevall, 2017). (iii) Melanins scatter radiation, causing it to be redirected in different directions and reducing its intensity upon contact. The physical shielding capacities of 0.5 mg/mL of solutions of Pyo_Fun_ and Pyo_Syn_ against UV-C radiation were determined to be 81.13 ± 0.27 J/m^2^ (*n* = 6), whereby the solvent itself (0.1 M KOH) was responsible for 19.40 ± 0.31 J/m^2^ (*n* = 6).

**Figure 8:**
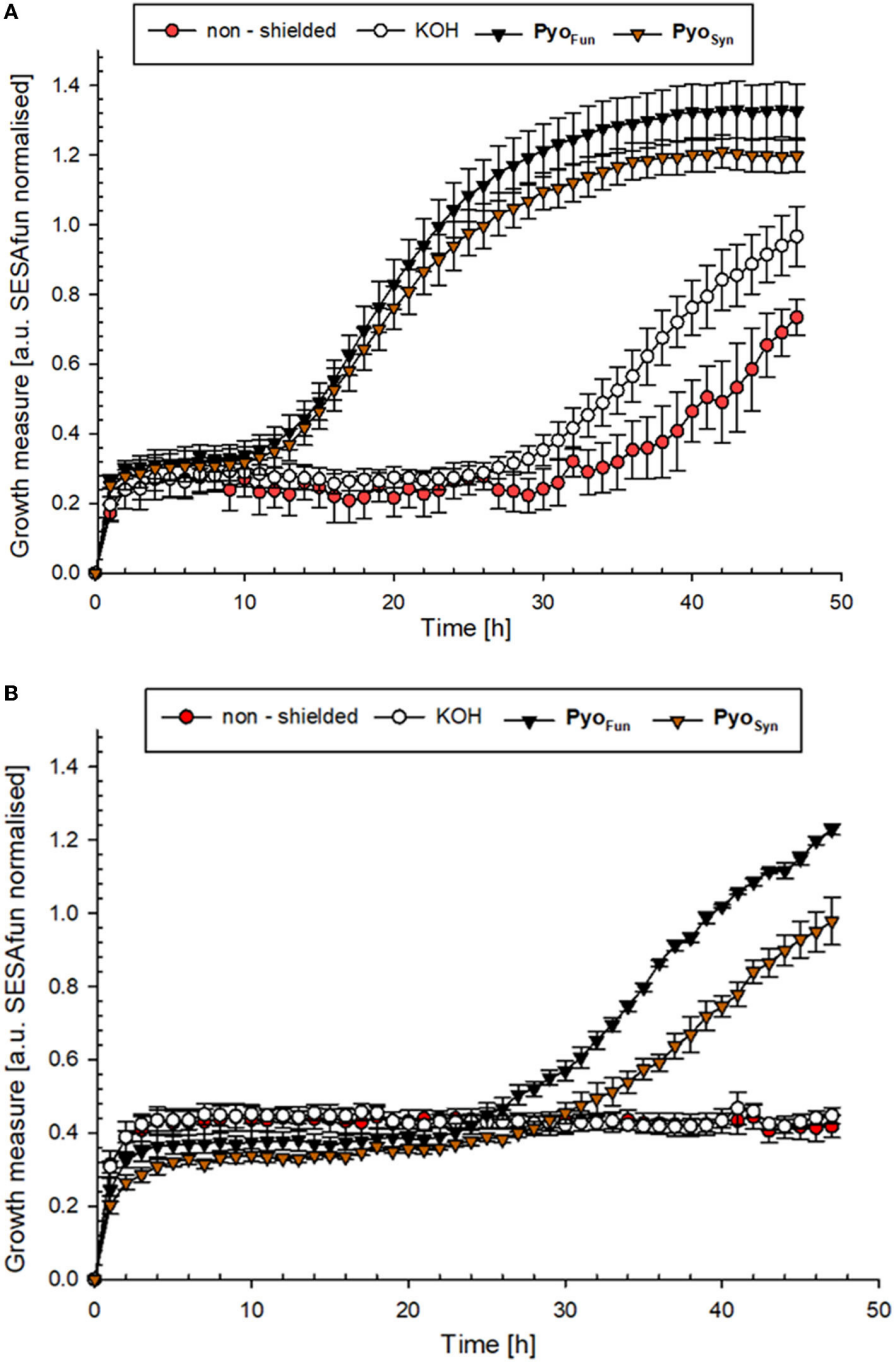
UV-C protection potential of Pyo_Fun_ and Pyo_Syn_. **(A)** Growth curve of *A. niger* strain N402 (wild type) and **(B)** growth curve of *A. niger* strain MA93.1 (Δ*fnwA*) after exposure to UV-C radiation. Measurements were performed in six biological replicates.

To determine the biological shielding capacity of Pyo_Fun_ and Pyo_Syn_ against UV-C radiation, we subjected spores of an *A. niger* wild-type strain (N402) and spores of a strain deficient for *fwnA* and thus DHN-melanin formation (MA93.1), (Jørgensen et al., 2011) to a UV-C dose rate of 1,038 J/m^2^. We choose this dose rate because it corresponds to the LD_90_ for strain N402 (Cortesão et al., 2020a). Importantly, before we treated the spores with UV-C, each spore monolayer was protected with a corresponding quartz glass cuvette filled with 0.5 mg/ml Pyo_Fun_, Pyo_Syn_ dissolved in 0.1 M KOH. As a reference, we used a quartz glass cuvette filled with only 0.1 M of KOH was used. Spore survival was followed by a live microscopic recording of spore germination and mycelial formation 48 h post-radiation (Figure 8), and survival rates were also calculated by determining colony forming units 48 h post-radiation (Table 2).

**Table 2:** Survival rates of *A. niger* spores when shielded with pyomelanin against UV-C (254 nm).

**Condition**	**Survival rates for wild type**	**Survival rates for *ΔfnwA***
0.5 mg/ml of Pyo_Fun_	0.71 ± 0.12 *p* = 0.280	0.98 ± 0.3^**^p = 0.007
0.5 mg/ml of Pyo_Syn_	0.15 ± 0.03 *p* = 0.068	0.54 ± 0.24 *p* = 0.089
0.1 M KOH	0.13 ± 0.07 *p* = 0.408	0.02 ± 0.008 *p* = 0.764
Non-shielded	0.06 ± 0.01	0.01 ± 0.003 *p* = 0.012 (wild type vs. mutant)

Spores of strains N402 (wild type) and MA93.1 (Δ*fnwA*) were subjected to a UV-C dose 1,038 J/m^2^ and thereafter allowed to germinate and grow on MM agar plates for 2 days. Colony-forming units (CFUs) were counted, and survival rates were calculated (*n* = 6). Initial spore concentration used was 10^6^/ml and pyomelanin dissolved in 0.1 M KOH (see Materials and methods section). The survival rates were normalized to N0 and *p*-values are all calculated in comparison to the non-shielded control, correspondingly. The significance threshold of the *p*-value for all statistics performed was 0.05, using a 95% two-tailed *t*-test for the difference of means.

The growth curves shown in Figure 8 demonstrated that both strains were efficiently protected by Pyo_Fun_ and Pyo_Syn._ Although, the trajectories of the growth measure curves in Figure 8 show that the DHN-melanin-deficient strain had a longer recovery phase after irradiation before the onset of germination (30 h of incubation) than the wild-type strain (10 h of incubation).

Comparing the survival rates of the unprotected wild-type strain N402, the DHN-melanin-deficient strain MA93.1 survived significantly less radiation (*p* = 0.012) (Table 2), as expected, and reported earlier (Cortesão et al., 2020a). However, we see that fungal pyomelanin contributed to the radioresistance of *A. niger*, as also reported earlier (Cortesão et al., 2020a). Especially in the DHN-melanin-deficient strain MA93.1, the radioprotection effect of Pyo_Fun_ was significantly higher compared with the non-shielded control (*p* = 0.007) and the KOH solvent control (*p* = 0.021). Interestingly, the radioresistance effect of Pyo_Fun_ was higher for the mutant strain than for the wild type. In this study, the shielding of Pyo_Fun_ was not statistically significant (*p* = 0.280) compared with the non-shielded control. In contrast, Pyo_Syn_ did not show a significant protective effect in any of the exposed strains.

## 4. Discussion

Conventional radiation shielding materials are generally limited in their protective efficiency as well as their versatility toward space radiation which consists of not only one but various types of particles, including high-energy cosmic rays and solar particle events. Traditional radiation shielding materials, such as lead or polyethylene, are not suitable for shielding applications for space travel. They impose structural challenges that are not compatible with future spacecraft's design and construction requirements (multifunctional, regenerative, and lightweight) which make them impractical for space travel due to weight launch constraints (Dadachova et al., 2008).

Melanin, on the other hand, is a lightweight, biocompatible, and versatile material that can be produced in large quantities *in vitro* (Vasileiou and Summerer, 2021). The biotechnologically harnessed cell factory *A. niger* bears exciting potential to be used in future space missions. As a multi-purpose cell factory that produces manifold molecules and compounds (Cairns et al., 2018), it can play a significant role in space biotechnology. Following the ability of *A*. *niger* to produce conservatives, proteins, enzymes, and antibiotics, we showed in this study that it is naturally capable of producing and secreting the secondary metabolite pyomelanin on a g/L scale. This kind of melanin could potentially be used to protect humans, materials, and living habitats on the Moon or Mars against cosmic and solar radiation. Furthermore, as melanins are also known to react with and bind metals (Cordero and Casadevall, 2017), the ability of *A. niger* to produce (pyo)melanin could also be harnessed in environmental protection and remediation efforts either on Earth or in space.

Many different routes have so far been researched to produce pyomelanin including chemical, enzymatic, and microbial approaches, with the production in unicellular fungi (*Yarrowia lipolytica*) or multicellular fungi (*A. niger*) being superior (Table 3). We envision a large optimization potential for *A. niger*, as the strain established in this study (OS4.3) has not yet been fully genetically and metabolically optimized (e.g., via overexpression of pyomelanin pathway genes). Moreover, much room for increasing titer and yield will be possible through process engineering as we have shown earlier for *A. niger* producing secondary metabolites of the cyclodepsipeptide type (Richter et al., 2014).

**Table 3:** Established pyomelanin production routes.

**Approach**	**Description**	**Titer/yield**	**Source**
Chemical production	Autooxidation of precursor molecule homogentisic acid	0.317 g per g of precursor molecule	(Lorquin et al., 2021, 2022)
Production in yeasts	*Yarrowia lipolytica* grown on optimized medium *Yarrowia lipolytica* mutant strain grown on optimized medium	0.5 g per l of medium 4.5 g per l of medium	(Tahar et al., 2020; Larroude et al., 2021)
Production in bacteria	*Pseudomonas putida* mutant strain grown on optimized medium *Halomonas titanicae* grown on optimized medium	0.35 g per l of medium 0.55 g per l of medium	(Nikodinovic-Runic et al., 2009; Lorquin et al., 2021)
Enzymatic production	Recombinant laccase enzyme with precursor molecule	1.25 g per g of precursor molecule	(Lorquin et al., 2021)
Production in filamentous fungi	*Aspergillus niger* inactivated for homogentisic acid degradation	1.48 g per l of medium	This study

To characterize the pyomelanin produced by *A. niger* (Pyo_Fun_), we performed distinct spectroscopic analyses (UV-Vis, FTIR, Raman) and validated its antioxidant and UV-C shielding capacity. All comparative spectroscopic analyses of Pyo_Fun_ and Pyo_Syn_ unambiguously confirmed the identities of the pigments as pyomelanin. Interestingly, purified Pyo_Fun_ scavenges more free radicals than the culture supernatant from strain OS4.3 (Figure 7), suggesting that purification after cultivation is important for achieving high ROS scavenging activities. To the best of our knowledge, no fungal, bacterial, chemical, or enzymatic-derived form of pyomelanin was tested so far for its photostability against monochromatic UV-C 254_nm_, which is one majorly harmful form of UV light in space. Notably, we could show that already a small amount of dissolved Pyo_Fun_ (0.5 mg/mL) shielded the melanin-deficient strain MA93.1 significantly against the LD_90_ UV-C dose of the fully pigmented wild-type strain N402. The protective effect was higher for the melanin-deficient strain, which was expected, due to its higher susceptibility toward radiation. We additionally took notice that the protective effect of Pyo_Fun_ seemed to be slightly better compared with the synthetic Pyo_Syn_ (e.g., compare data in Figure 7), however, if such a difference exists remains to be shown in future studies. Moreover, it will be of interest in future studies to understand whether and how Pyo_Fun_ and Pyo_Syn_ can be applied in a dried form since natural melanin pigments are mostly deposited in skin/tissue/cells in the form of spherical granules (Castellano-Pellicena et al., 2021) clusters. In future, we will also extend our analyses toward the potential radioprotective capabilities of Pyo_Fun_ against X-ray and cosmic radiation and to better understand how physical and biological shielding of *A. niger's* pyomelanin can be mechanistically explained.

## 5. Conclusion

In conclusion, the generated recombinant mutant strain OS4.3 of *A. niger*, capable of extracellular overproduction of pyomelanin via the disruption of the *hmgA* gene, has shown very promising results. With presented spectroscopic and chemical analysis methodologies, we characterized and identified the excessively secreted pigment as fungal pyomelanin. Although the production sequence for pyomelanin from OS4.3 has not yet been optimized for a large upscaled production, the average Pyo_Fun_ yield extracted from a 200-mL culture of OS4.3 has shown a good bioconversion rate from L-tyrosine as a precursor and in future a metabolic flow analysis will help in improving the upscaling process. The discovered scavenging effect of Pyo_Fun_ against reactive oxygen species and its shielding capacity against radiation for UV-C doses in the range of space environment (1,038 J/m^2^) provide a promising potential for bio-shielding applications. Although the pigments have only been assessed in dissolved form so far, further research is necessary to test them in a dried form, similar to how melanin pigments are found in nature. However, the findings of this study show that pyomelanin could be a valuable biologically derived agent for shielding human tissue and coat materials against harmful radiation, highlighting the significance of further exploration in this area.

## Data availability statement

The original contributions presented in the study are included in the article/Supplementary material, further inquiries can be directed to the corresponding authors.

## Author contributions

VM and RM conceived the study. CP and OS designed molecular work in *A niger* and generated and genetically verified the strains. SK, AM, MC, and OS cultivated, extracted, and purified the pigments. SK designed and performed antioxidant and UV-C shielding assays, the UV-Vis spectroscopic analyses, and the FTIR spectroscopic analyses with the help of Gesellschaft für Werkstoffprüfung MBH. KRe designed and performed Raman spectroscopic analyses. All authors were involved in data interpretation and manuscript writing.
