# Supplementary material for: Aspergillus niger as a cell factory for the production of pyomelanin, a molecule with UV-C radiation shielding activity
Source: Front Microbiol. 2023 Jul 20;14:1233740. doi: 10.3389/fmicb.2023.1233740 (PMC10399693; doi:10.3389/fmicb.2023.1233740)

Supplementary Material

**Table SI 1 -** Protospacers and primers used in this study

| **Name** | **Sequence (5’ – 3’)** | **function** |
| --- | --- | --- |
| protospacer-T44_hmgA | ACTAGGTCGGAGGCTATCAAGGG | Target of Cas9 in hmgA, PAM underlined |
| protopacer_T50_hmgA | ACTGGCCCGTCCGATCACAACGG | Target of Cas9 in hmgA, PAM underlined |
| protospacer-T24_hppD | TTACGCGCATGGGTTTCAAGCGG | Target of Cas9 in hppD, PAM underlined |
| protopacer_T34_hppD | TCAGGTGGTTAACATACTATTGG | Target of Cas9 in hppD, PAM underlined |
| sgRNA-control-Primer-1 | GCTACCATCATTTCCCATCAAATAGG | Validation of sgRNA integration into pFTK086 |
| hmgA-KO-73bp-fw | TAACCTCCTTGGCTCAGAAGTGCTTGTGCAGTTAAACGGTCACCAAGACTGAAGCACTGAACCACTACCCACCCACATTGCGGACGTTTTTAATGTACTG | amplification of dDNA with short homology arms for deletion of hmgA |
| hmgA-KO-74bp-rev | TATGATTGAATACAACTACTACATAGGTATCTGAAAAATCACCTTCGGTTTTAGTAACTTCGTCGGATCGGTGTCCGCCAATATATCCTGTCAAACACTG | amplification of dDNA with short homology arms for deletion of hmgA |
| hppD-KO-73bp-fw | GACATCTCCCTTCTATACCCAAGTATTATAGAATCTCTTTGCTGTTTTTCATACAACATACAACTCCATCACCCACATTGCGGACGTTTTTAATGTACTG | amplification of dDNA with short homology arms for deletion of hppD |
| hppD-KO-74bp-rev | GCAACCTGGCCGAATGTACACTATCCCCTTCAAATAACTGTCTTGCAGTTCTGTACTGAGAAAATAAATAAAACCCGCCAATATATCCTGTCAAACACTG | amplification of dDNA with short homology arms for deletion of hppD |
| cPCR_hmgA_f1 | GCGATGCCGACGTTAAAAACGAATC | diagnostic PCR of hmgA deletion |
| cPCR_hmgA_r1 | TAGGTGCTTTGGGAGTGTGCTTTTC | diagnostic PCR of hmgA deletion |
| cPCR_hppD_f1 | ACTGCCTGTGCTTGCTTATCCTC | diagnostic PCR of hppD deletion |
| cPCR_hppD_r1 | TCCCATGATGATCCCAACTTACT | diagnostic PCR of hppD deletion |
| An15g00560_act1_fw | ACAATGAACTCCGTGTCGCT | qPCR on An15g00560 (actin) |
| An15g00560_act1_rev | GATGGAGACGTAGAAGGCGG | qPCR on An15g00560 (actin) |
| An01g00530-pepA_fw | ACATCCCTGGTTCCGACAAG | qPCR on An01g00530-pepA |
| An01g00530-pepA_rev | GATCCAGCAGAGACACTGGG | qPCR on An01g00530-pepA |
| An15g00610-hisB_fw | TCCTAATCGCTACCGCCAAC | qPCR on An15g00610-hisB |
| An15g00610-hisB_rv | TCACGCATGGATTGCAGAGT | qPCR on An15g00610-hisB |

**Table SI 2 –** Comparison of *A. niger* and *A. fumigatus* genes, including homology on the protein level

| **Gene** | **Gene ID A. fumigatus** | **Gene ID A. niger** | **Identity (protein)** | **function** |
| --- | --- | --- | --- | --- |
| *hppD* | Afu2g04200 | **An11g02200** | **85 %** | dioxygenase |
| *hmgA* | Afu2g04220 | **An11g02180** | **93 %** | dioxygenase |
| *maiA* | Afu2g04240 | **An11g02160** | **70 %** | isomerase |
| *fahA* | Afu2g04230 | **An11g02170** | **84 %** | hydrolase |
| *hmgX* | Afu2g04210 | **An11g02190** | **66 %** | hypothetical protein |
| *hmgR* | Afu2g04262 | **An11g02150** | **62 %** | transcription factor |


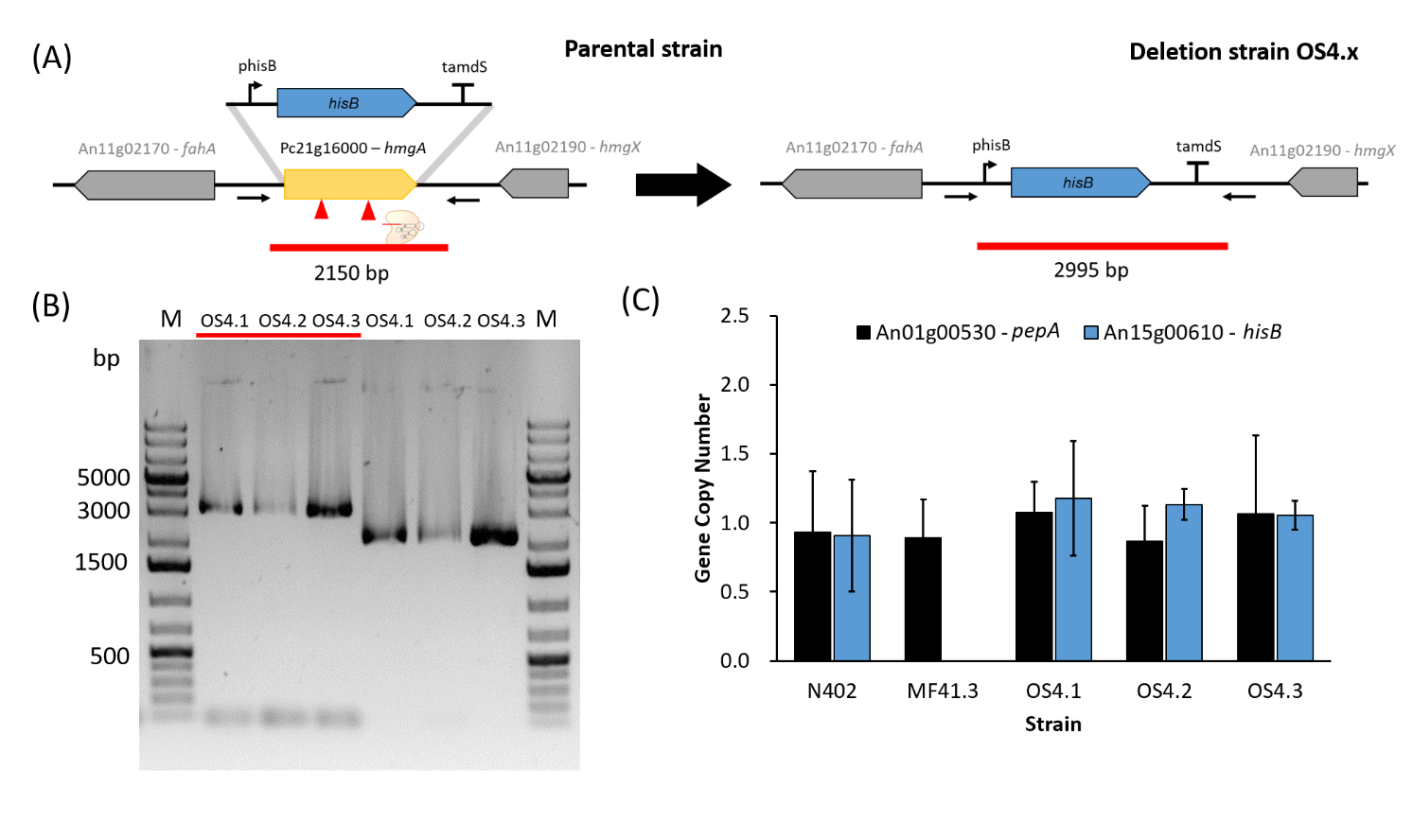


**Figure SI 1 – Verification of *hmgA* deletion strains. (A**) Scheme of expected PCR product sizes in parental strain and in KO strains. Protospacers for Cas9 are indicated by red triangles. **(B)** Results of PCR using primers cPCR_hmgA_f1 and cPCR_hmgA_r1 on 3 randomly selected transformants (lane 2 to 4), expected size: 2043 bp. Lane 5 to 7 – control of PCR success using cPCR_ hppD _f1 and cPCR_hppD_r1. Ladder is 1kb plus DNA Ladder (Thermo Scientific). **(C)** Results of gene copy number determination by quantitative PCR.


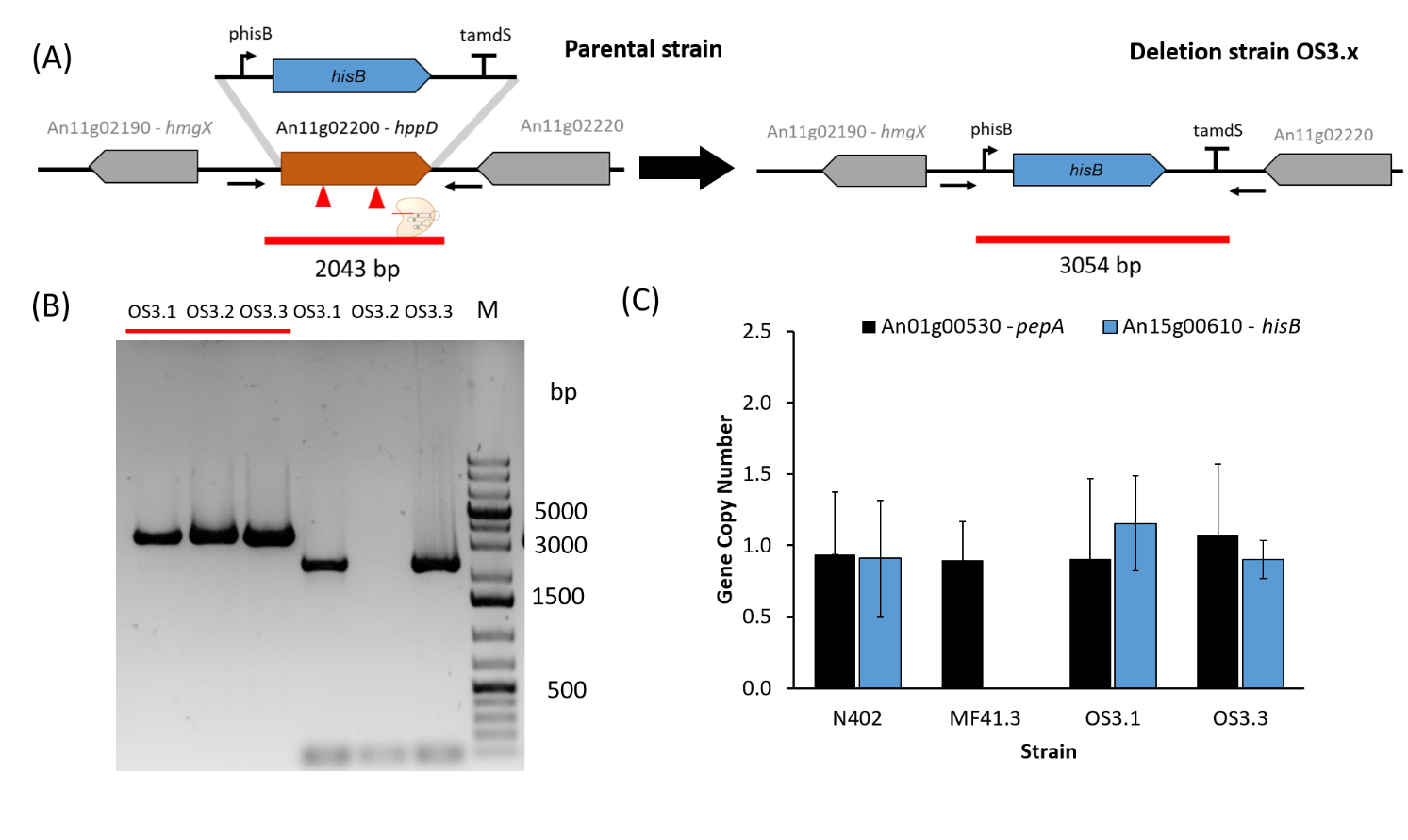


**Figure SI 2 – Verification of *hppD* deletion strains.** (A) Scheme of expected PCR product sizes in parental strain and in KO strains. Protospacers for Cas9 are indicated by red triangles. (B) Results of PCR using primers cPCR_hppD_f1 and cPCR_hppD_r1 on 3 randomly selected transformants (lane 1 to 3) expected size: 3054 bp. Lane 4 to 5 – control of PCR success using cPCR_ hmgA _f1 and cPCR_hmgA_r1. Ladder is 1kb plus DNA Ladder (Thermo Scientific). (C) Results of gene copy number determination by quantitative PCR.


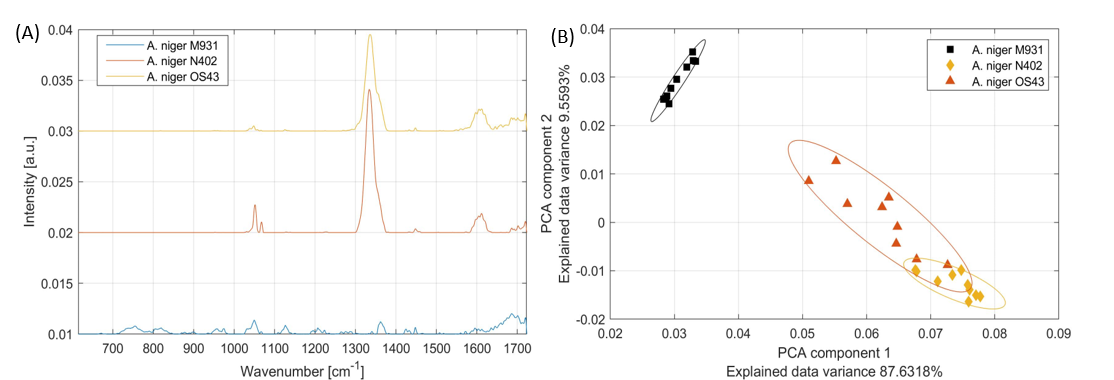


**Figure SI 3** (A) RAMAN Spectre overlay of *A. niger* wild-type (N402), pyomelanin production strain (OS 4.3) and a melanin synthesis deficient mutant strain (MA93.1). (B) PCA (Principal Component Analysis) showing data variance of all three strains shown in (A).

**Figure SI 4 Experimental set-up for testing shielding capacities** (A) Exemplary overview on irradiation source (UV-Lamp and dosimeter) (B) Exemplary close – up on dosimeter sensor covered with filled quartz – glass cuvette (C) Exemplary close – up on 96 – well plate (containing spores for irradiation) covered with different shielding conditions.


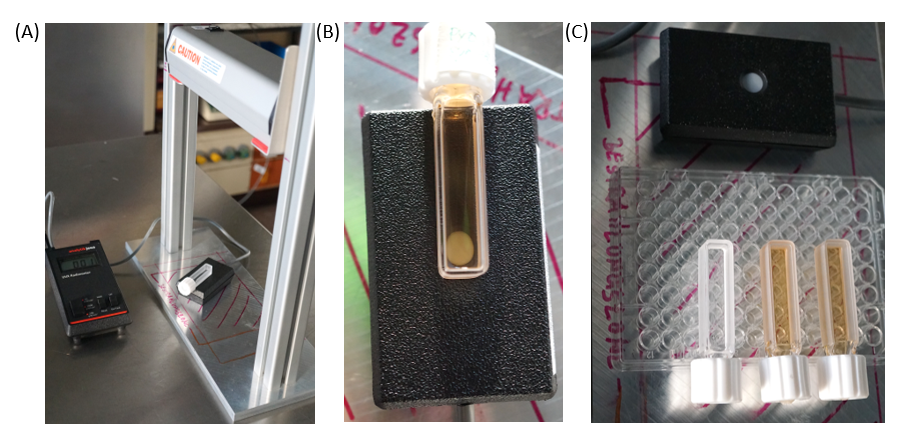

Supplement: Supplementary file 1 [file Data_Sheet_1.docx]
